# Gold-Catalyzed Cyclizations of Alkynol-Based Compounds: Synthesis of Natural Products and Derivatives

**DOI:** 10.3390/molecules16097815

**Published:** 2011-09-13

**Authors:** Benito Alcaide, Pedro Almendros, José M. Alonso

**Affiliations:** 1 Grupo de Lactamas y Heterociclos Bioactivos, Departamento de Química Orgánica I, Unidad Asociada al CSIC, Facultad de Química, Universidad Complutense de Madrid, 28040-Madrid, Spain; 2 Instituto de Química Orgánica General, CSIC, Juan de la Cierva 3, 28006-Madrid, Spain

**Keywords:** gold catalysis, alkynols, total synthesis, natural products

## Abstract

The last decade has witnessed dramatic growth in the number of reactions catalyzed by gold complexes because of their powerful soft Lewis acid nature. In particular, the gold-catalyzed activation of propargylic compounds has progressively emerged in recent years. Some of these gold-catalyzed reactions in alkynes have been optimized and show significant utility in organic synthesis. Thus, apart from significant methodology work, in the meantime gold-catalyzed cyclizations in alkynol derivatives have become an efficient tool in total synthesis. However, there is a lack of specific review articles covering the joined importance of both gold salts and alkynol-based compounds for the synthesis of natural products and derivatives. The aim of this Review is to survey the chemistry of alkynol derivatives under gold-catalyzed cyclization conditions and its utility in total synthesis, concentrating on the advances that have been made in the last decade, and in particular in the last quinquennium.

## 1. Introduction

Organic synthesis has as one of its major points of interest the study of naturally occurring substances, and this remains both a source of information and an intellectual challenge. Thus, a crucial target for organic chemists is to find the appropriate reaction conditions, allowing functional group compatibility and providing high efficiency and atom economy. During the last years, gold-catalyzed cycloisomerization of alkynol-based systems has emerged as a useful tool in this area, allowing the synthesis of different structures such as furans, dihydrofurans, pyrans, furanones or ketals, among many other heterocyclic systems and naturally occurring structures [1,2,3].

This overview focuses on the most recent achievements in gold-catalyzed cycloisomerization reactions, for the synthesis of natural products and related compounds. In particular, carbon-carbon and carbon-heteroatom cyclization processes will be considered, paying special attention to reports from the last five years.

## 2. Cycloisomerization Processes Involving Carbon-Carbon Bond Formation

Gold-catalyzed cycloisomerization reactions involving C−C bond formation have recently emerged as an effective methodology to build hydrocarbon rings. Four, five and six membered cyclic structures, as well as medium sized rings are accessible in good yields and under interesting mild reaction conditions using gold salts and gold complexes. Fused bicyclic compounds can also be produced, leading therefore to an attractive series of natural occurring skeletons.

**Figure 1:**
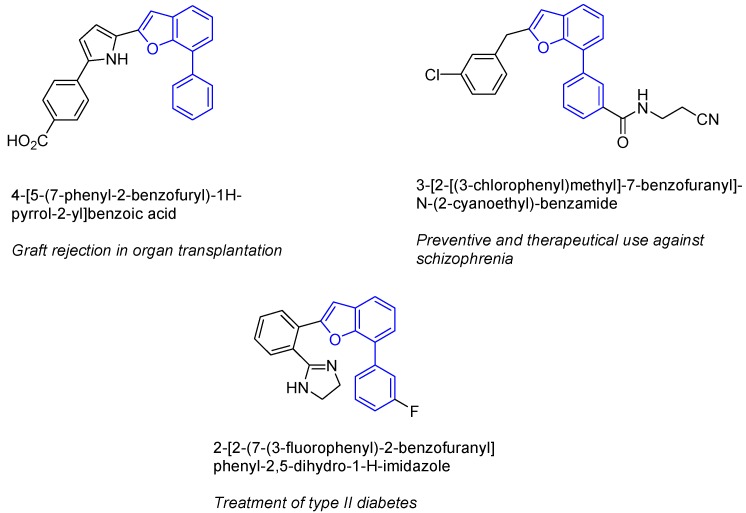
7-Aryl benzofuran structure core in different bioactive compounds.

Benzofurans represent a recurring motif among natural products. Particularly, 2-substituted and 2,7-disubstituted benzofurans and their derivatives are known to show many different biological activities such as antineoplastic, antiviral, antioxidative or anti-inflammatory properties. Although many routes for the preparation of 2-substituted systems have been developed [4,5], 2,7-substituted benzofurans remain almost unexplored (Figure 1) [6,7,8].

**Scheme 1:**
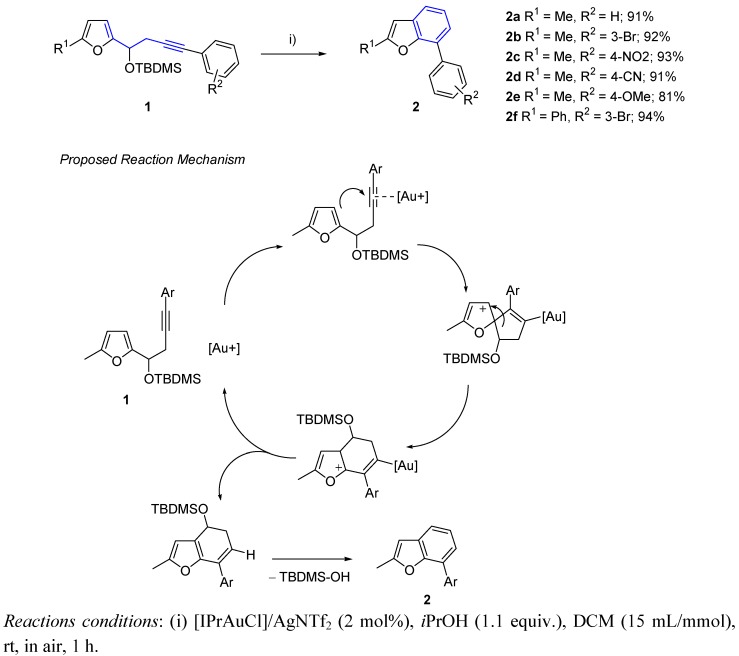
Gold(I)-catalyzed rearrangement of 3-silyloxy-1,5-enynes.

Hashmi *et al*. have recently proposed an efficient route leading to 7-aryl benzo[b]furans **2** through a gold-catalyzed rearrangement of 3-silyloxy-1,5-enynes [9]. The considerable effort that went into this work, involving a first catalyst screening for substrate **1a** and finding the optimal conditions for the dual catalyst system [IPrAuCl]/AgNTf_2_ is noteworthy. Thus, an easy methodology was performed, using mild conditions, open-air systems and remarkable short reaction times, providing an interesting family of different substituted 7-aryl benzofurans (Scheme 1).

**Figure 2:**
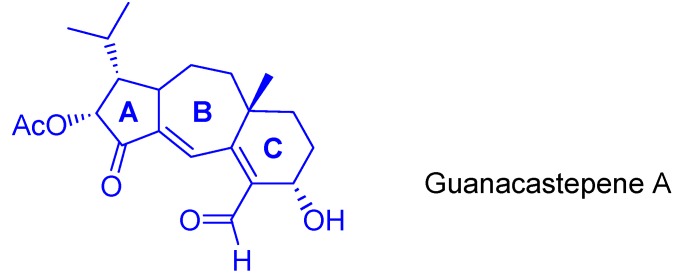
Rings A, B and C in natural terpene guanacastepene A.

Approximately a quarter of biologically active known compounds come from fungi, and among their wide range of properties, the antibiotic activity has attracted much interest [10,11]. Guanacastepene A (Figure 2) is extremely active against methicillin-resistant strains of *Staphylococcus aureus* and vancomicyn-resistant *E. faecalis*, two drug-resistant common pathogens which have generated major concern [12,13,14].

**Scheme 2:**
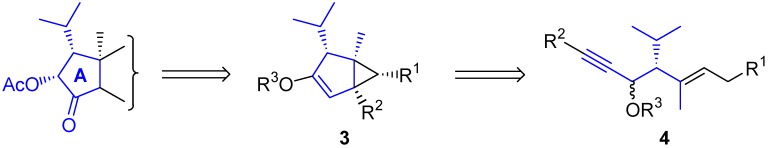
Retrosynthesis of guanacastepene ring A.

It has been stated an approach to ring A of guanacastepene, by an unusual gold(I)-catalyzed cycloisomerization of alkynol-based 1,5-enynes [15]. According to the proposed retrosynthesis, most of the functionalities present in the natural terpene would be early introduced, while the presence of the cyclopropyl fused ring could allow the further generation of ring B (Scheme 2).

**Scheme 3:**
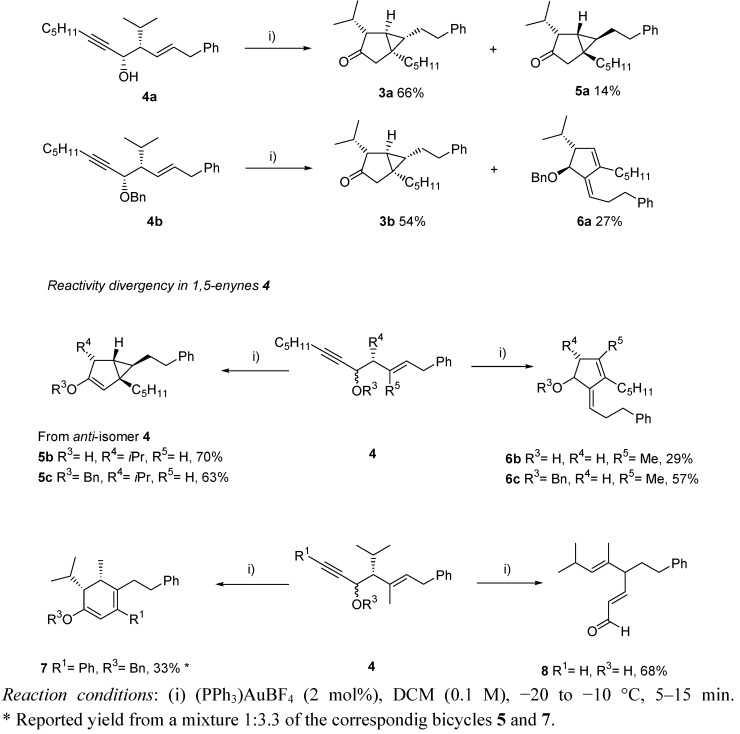
Divergent reactivity for the gold-catalyzed reaction of 1,5-enynes.

Many 1,5-enynes were tested, and an unexpected pattern of reactivity depending on the substituents in substrates **4** was found. Thus, the desired bicyclo[3.1.0] system **3** was obtained only when the reaction was performed with the *syn*-enynes **4a** and **4b**, yielding **3a** and **3b** with good conversions and notable diastereoselectivity. *Anti*-isomers, or any stereochemical change on the starting 1,5-enynes, resulted in the opposite diastereoselectivity (systems **5**), or a dramatic change on the course of the reaction, leading to alkylidene-cyclopentenes **6**, cyclohexadienes **7**, or α,β-unsaturated aldehydes **8** (Scheme 3).

**Scheme 4:**
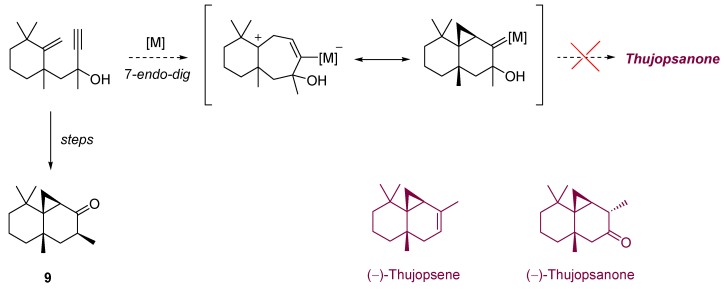
Projected synthetic route to terpene (−)-thujopsanone.

**Scheme 5:**
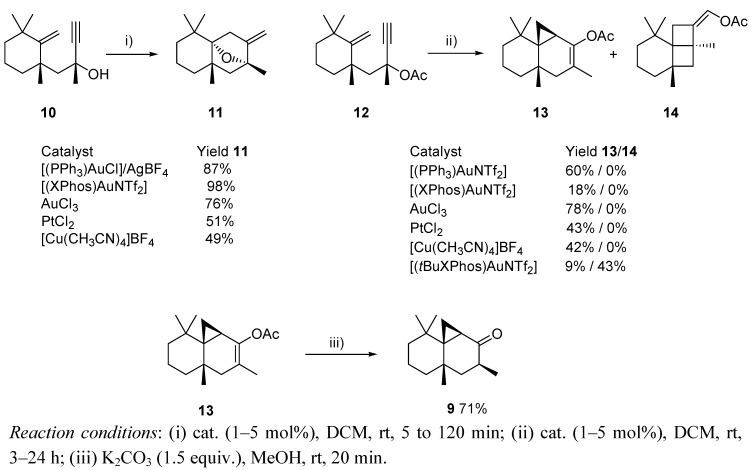
Cycloisomerization of enynol **10** and [1,2]-acyl shift rearrangement of acetate **12**, respectively.

Gold-catalyzed isomerization has been also employed in the search of an appropriate route to (−)-thujopsanone, a derivative of the natural terpene (−)-thujopsene, widely employed in cosmetics [16]. Although the first aim of the authors remained unachieved, and the obtained compound **9** did not exhibit the appreciated properties of the initial target [17], the chemistry developed merits further consideration (Scheme 4) [18]. Thus, it was observed that enynol **10** provided the unexpected ether **11** in the presence of different gold catalysts, in amounts similar to those produced by some other metal salts such as copper or platinum complexes. Interestingly, when the corresponding acetate derivative **12** reacted in the presence of AuCl_3_, a tandem cycloisomerization/[1,2]-acyl shift took place, leading to adduct **13**, precursor of the previously mentioned adduct **9**, and a close system to (−)-thujopsanone. Moreover, when the process was tested in the presence of (*t*BuXPhos)AuNTf_2_ as catalyst, an unprecedented rearrangement/cycloaddition leading to the tricyclic system **14** was reported (Scheme 5).

**Figure 3:**
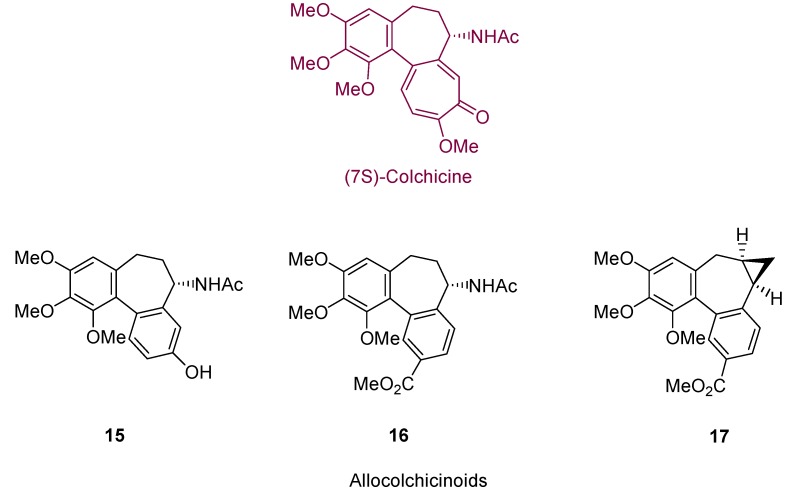
Colchicine and allocolchicinoids systems.

Gold-catalyzed cycloisomerization methodology has also been applied to the construction of medium sized rings. Allocolchicinoids, presenting a seven membered ring, are structures related to (−)-colchicine, a natural product with important antimitotic activity (Figure 3). Many of these derivatives also show this kind of mitosis arrest, by inhibiting tubulin polymerization [19,20,21]. *N*-acetylcolchinol **15**, for instance, is described to bind to tubulin more strongly than colchicine itself. Thus, many reports have appeared describing the synthesis of these structures [22,23,24,25,26,27,28].

**Scheme 6:**
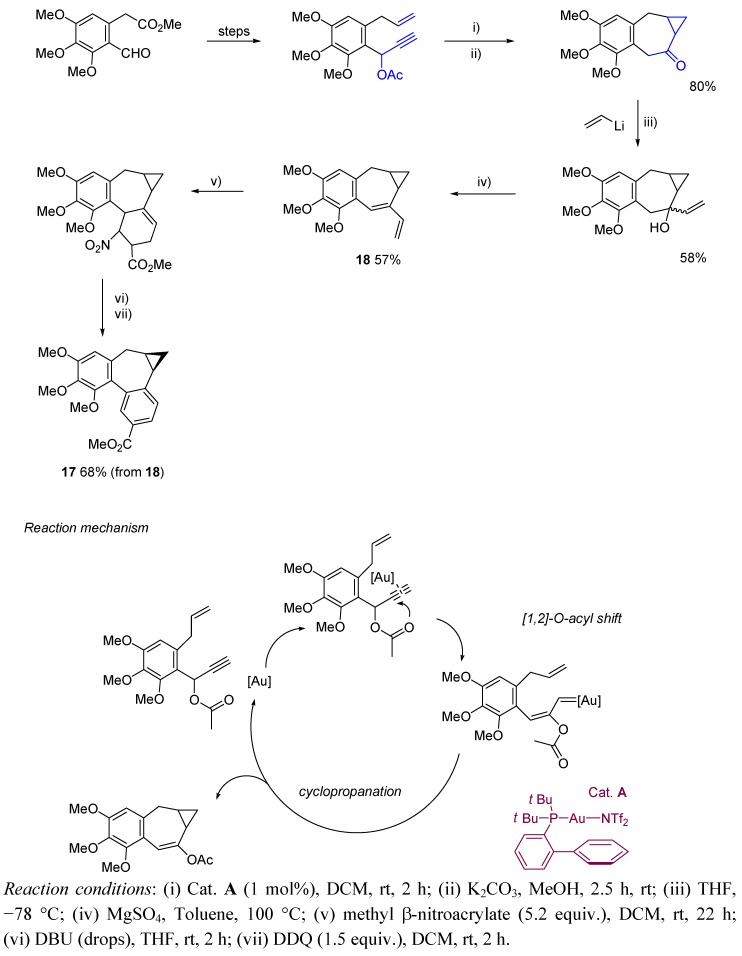
Synthesis of allocolchicinoid **17** and proposed mechanism for the gold-catalyzed cycloisomerization step.

Hanna *et al.* reported the synthesis of derivative **17** [29]. In the proposed sequence, the seven membered ring is formed by a gold(I)-catalyzed 1,2-*O*-acyl shift, followed by a cyclopropanation step which leads to the fused three member ring (Scheme 6). Thus, gold-catalyzed cyclization of alkynol-based systems has also been stated in this work as a useful tool to create medium sized rings, through an easy methodology providing high yields under mild reaction conditions.

Indole systems are ubiquitous in Nature, appearing in many different alkaloid families. Their wide range of biological activities, and their intriguing chemistry, makes these compounds a target of special interest, and a recurring topic in many studies [30,31,32,33,34,35,36,37,38,39,40,41]. For instance, the first enantioselective approach to (−)-mersicarpine, an alkaloid isolated from Kopsia plants and exhibiting an unusual tetracyclic structure has been reported. The proposed retrosynthetic analysis included the reaction of an alkynol-based intermediate in the presence of a gold salt, although only the alkyne functional group showed reactivity under these conditions, preserving the hydroxylic group for a further oxidation [42].

More interestingly, the reactivity of alkynol-based systems as formal organic synthons has been also explored in the indole chemistry. It has been established the synthesis of the non-natural skeleton 2,3-indoline-fused cyclobutane through a cascade process, including both C−C and C−O bond formation catalyzed by the same gold salt [43].

**Scheme 7:**
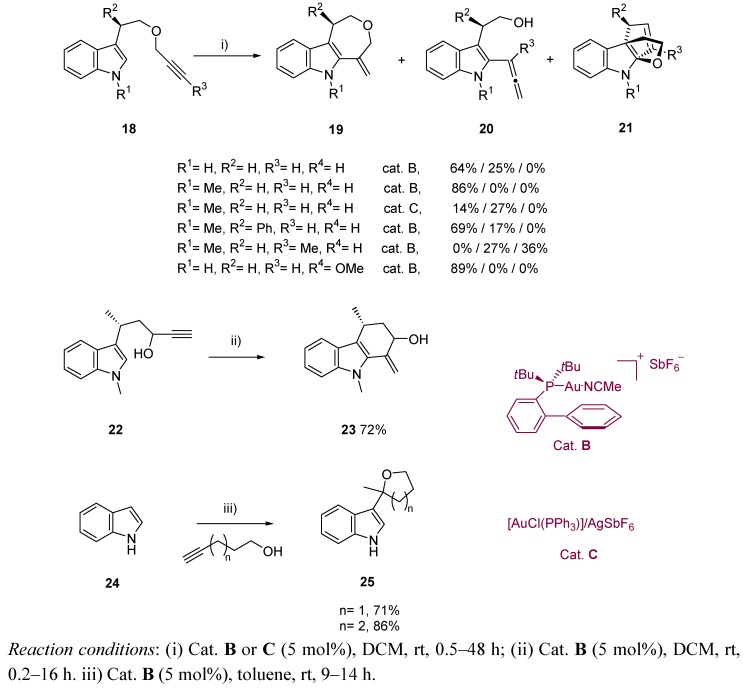
Alkynol-based reactivity in indole chemistry.

On the other hand, Echavarren *et al.* described in an exhaustive report about inter- and intramolecular gold-catalyzed reaction of alkynes and indoles some examples starting from alkynols and alkynol-based systems. Carbazole-like systems and related structures were therefore achieved (Scheme 7) [44].

**Scheme 8:**
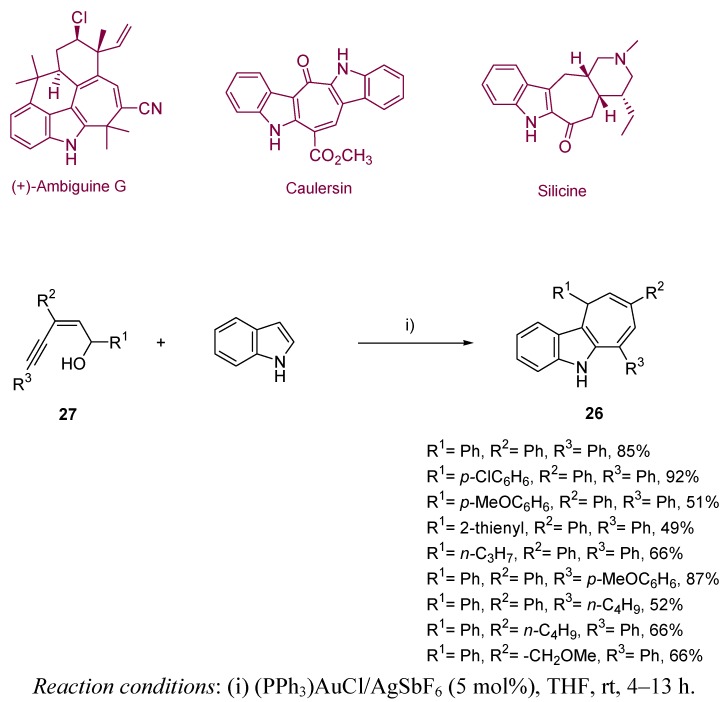
Synthesis of dihydrocyclohepta[b]indoles, and related natural structures.

**Scheme 9:**
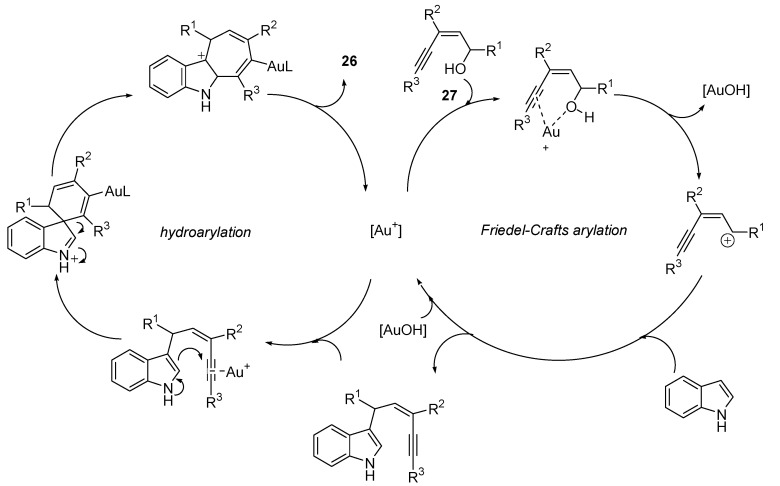
Proposed reaction mechanism for the tandem gold catalyzed-Friedel-Crafts arylation/hydroarylation process.

Inspired by the results of the Echevarren group, Liu *et al.* described the synthesis of dihydrocyclohepta[b]indoles **26** from (*Z*)-enynols **27** and indole, through an interesting domino sequence including a first gold(0)-catalyzed Friedel-Craft reaction, followed by a hydroarylation step [45]. The resulting products are of considerable interest, as much as they form the key subunits of several alkaloids, like ambiguine, silicine, caulerpin or caulersin. The reported work includes the optimization of the process, by testing different gold salts and solvents, leading to high reaction conversions through mild conditions (Scheme 8 and Scheme 9).

## 3. Cycloisomerization Processes Involving Carbon-Heteroatom Bond Formation

Heterocyclic natural occurring motifs such as furans, pyrans or spiroketals can be easily achieved through heterocyclization processes performed on alkynol-based systems. Gold promoted methodologies provide a convenient route to these structures, allowing mild reaction conditions and high yields. Total synthesis and the preparation of related derivatives have been recently described using both C−N and C−O bond formation.

### 3.1. Cycloisomerization on Alkynol-Based Systems

**Scheme 10:**
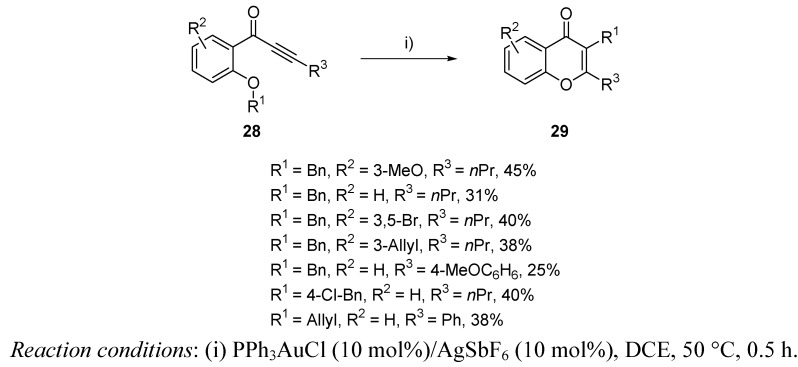
Synthesis of chromones by gold-catalyzed cycloisomerization.

Chromones are natural heterocycles showing a wide range of biological properties. Thus, many strategies like iodocyclizations [46], metal-catalyzed cycloadditions [47], or *O*-arylation processes [48] have appeared for the synthesis of these oxacyclic systems. Gold catalyzed cycloisomerization of alkynol based structures **28** have been also stated for the generation of chromones **29** [49]. Interestingly, reaction proceeded with a further migration of group R^1^, leading to highly functionalized skeletons. Unluckily, only moderate yields were achieved (Scheme 10), inasmuch as isomerization processes competed with the expected Au-based cycloisomerization.

**Scheme 11:**
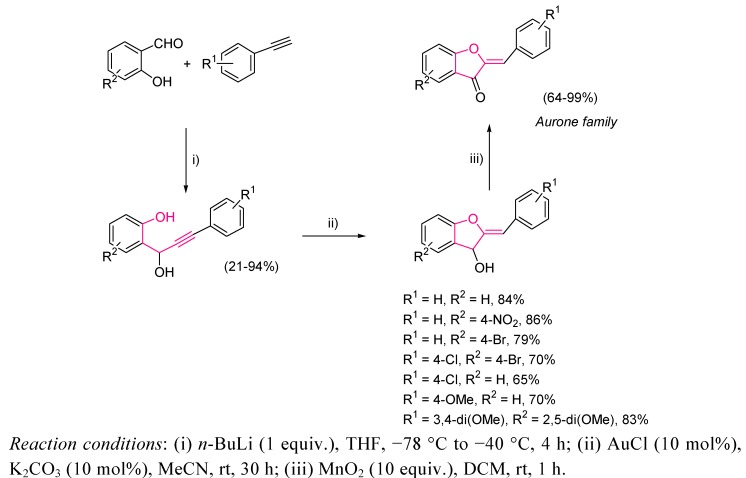
Synthesis of aurone skeleton by gold-catalyzed cycloisomerization.

A similar approach has been developed for the synthesis of aurone skeletons [50], natural flavonoids, by an easy three step sequence. Aurones exhibit several biological properties [51,52,53,54,55], and its importance had led to several groups to develop convenient synthetic routes [56,57,58,59,60,61,62]. Among them, gold-catalyzed oxycyclization provided the best results, as milder reaction conditions and excellent selectivities, avoiding the formation of flavones as byproducts, were achieved (Scheme 11) [63]. In this case, high yields and complete regioselectivity were obtained.

**Figure 4:**
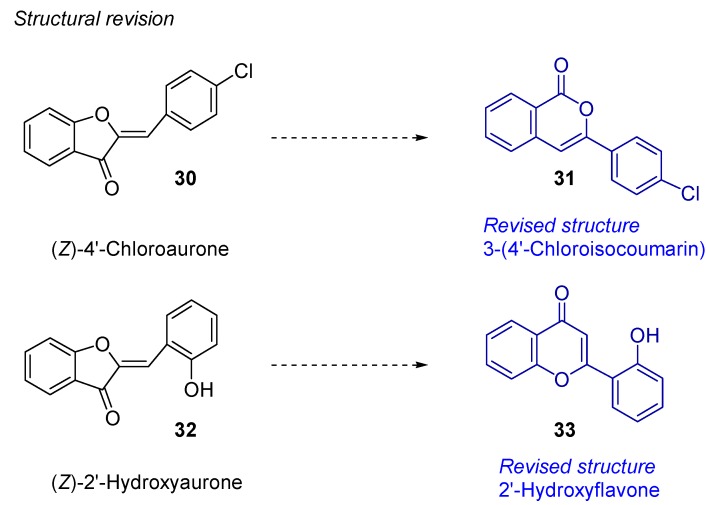
New assignation of structures **31** and **33** by comparison with the prepared by the gold catalysis aurone systems **30** and **32**.

Moreover, the present methodology was used for the structural revision of two natural products, (*Z*)-4’-chloroaurone **30** [64], and (*Z*)-2’-hydroxyaurone **32** [65], proving that the assumed structures were not the correct ones. Thus, flavonoid systems **30** and **32** could be prepared by the above three step strategy which revealed that their spectral data did not match with the previously reported data of the natural isolated ones. Therefore, the isocumarin **31** and the flavone **33** were prepared and probed as the real structures for these natural products (Figure 4).

**Scheme 12:**
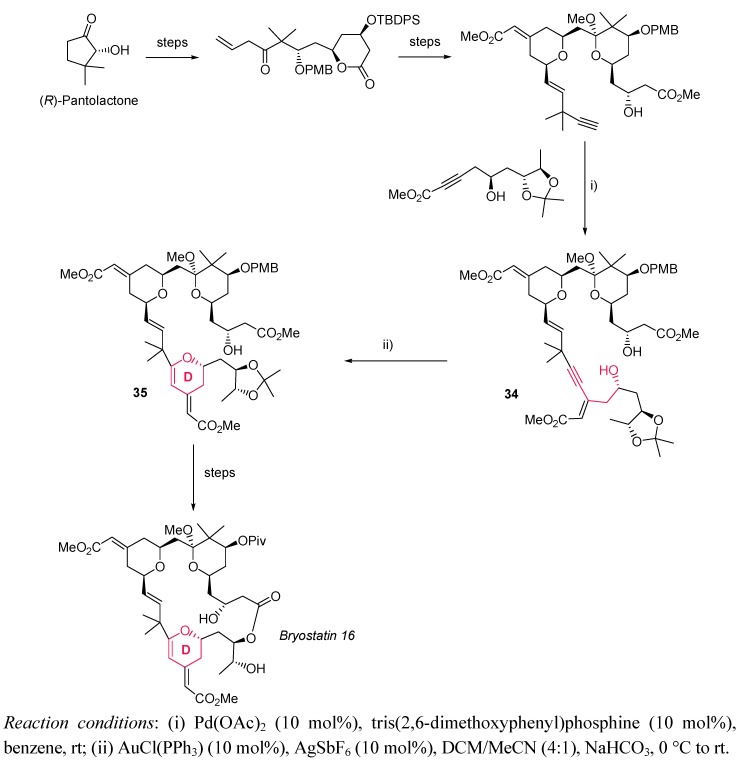
Gold-based synthesis of dihydropyran ring **D** in bryostatin total synthesis.

Trost *et al.* recently completed the total synthesis of bryostatin 16 [66,67], a structurally complex macrolide which exhibits a wide range of biological activities [68,69,70,71]. Focusing on the proposed 26 step sequence (in the longest linear path, and 39 steps as the total), the gold-catalyzed 6-*endo*-dig oxycyclization of alkynol **34** to generate the inner dihydropyran cycle **D** in macrocyclic precursor **35** in 65% yield deserves special attention (Scheme 12).

(+)-Cephalostatin 1 is another complex macrolide with interesting biological activity. It has been reported to be a promising anticancer agent for the *p16* tumor suppressor gene, exhibiting high activity and high selectivity between cancer cells and normal cells [72,73]. Because of the small amounts of cephalostatin available from its natural marine sources, a synthetic approach has emerged as the sole viable tool to provide enough material for biological testing [74,75,76,77,78]. On the other hand, the structural complexity of cephalostatin makes this macrocycle an interesting target to develop new skills in organic synthesis.

**Scheme 13:**
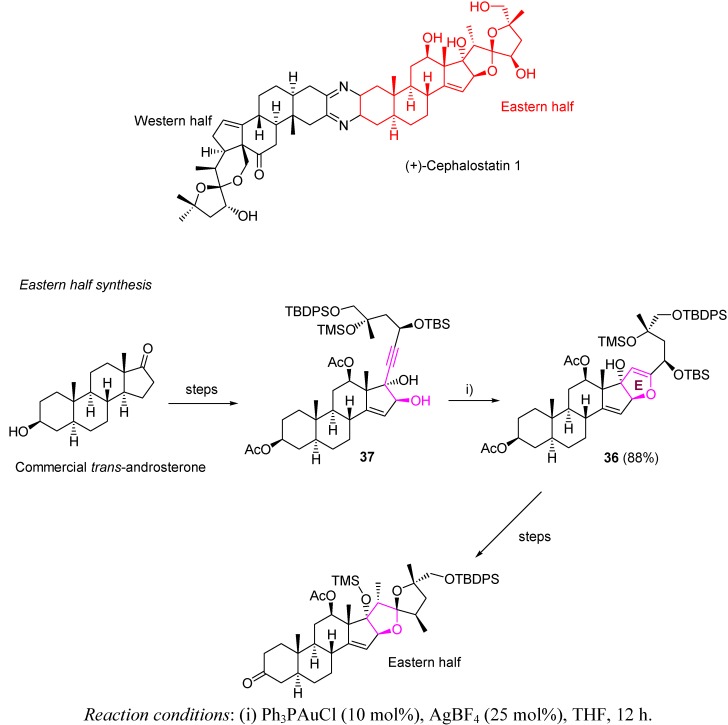
Synthesis of ring E on the eastern fragment of cephalostatin.

Fortner *et al.* have recently described a total synthesis of cephalostatin, involving the construction of both its eastern and western fragments and their further coupling [79]. Along the high quality chemistry developed for this synthesis, we would like to focus on the dihydrofuran ring **E** construction on compound **36**. Thus, gold-catalyzed cycloisomerization emerge again as a useful methodology to convert alkynol systems in oxacyclic skeletons, crucial and recurring motifs for total synthesis. Moreover, the efficiency of gold catalysis to promote a 5-*endo*-*dig* process with an 88% conversion, on what is a hindered internal alkyne **37**, deserves special consideration (Scheme 13).

**Scheme 14:**
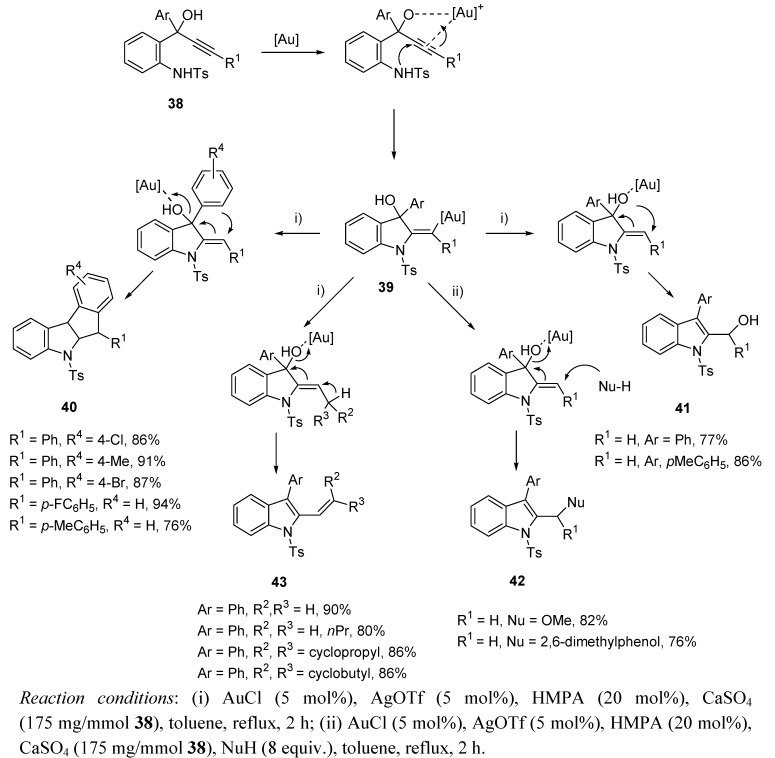
Indole synthesis from gold-catalyzed cycloisomerization of 2-tosylamino-phenylprop-1-yn-3-ols.

Other natural occurring motifs such as oxazoles and isoxazoles have also been assembled through gold-catalyzed cycloisomerization. Thus, it has been recently established a general method for the synthesis of highly functionalized isoxazoles from alkynyl oxime ethers [80], or an intermolecular alkyne oxidation leading to 2,5-disubstituted oxazoles [81]. Nevertheless, while gold-based alkyne-oxygen cycloisomerization has recently become a hot topic in organic synthesis, only a few examples for alkyne-nitrogen coupling have been described [82,83,84,85,86,87,88,89,90]. Regarding the synthesis of natural products and derivatives, Chan *et al.* have recently described the synthesis of highly substituted indole skeletons [29], from readily available 2-tosylamino-phenylprop-1-yn-3-ols **38** [91]. The reported work shows a versatile approach to these natural occurring motifs, and develops a fascinating study concerning the chemical reactivity of these substrates under gold-catalyzed conditions. Thus, starting in every case from a 5-*exo*-dig cycloaddition which led to vinyl gold species **39**, different reaction pathways were observed depending on the substituent group R^1^. It was stated that when R^1^ = aryl, reaction proceeded through a Friedel-Craft process, giving indenyl-fused indoles **40**. On the other hand, changing to R^1^ = H, a protodeauration/1,3-allylic alcohol isomerization took place, leading to indoles **41**. The presence of a nucleophile in the reaction media gave place mainly to systems **42**, and for R^1^ = CHR^2^R^3^, a more facile protodeauration and dehydratation step delivered systems **43** (Scheme 14).

**Scheme 15:**
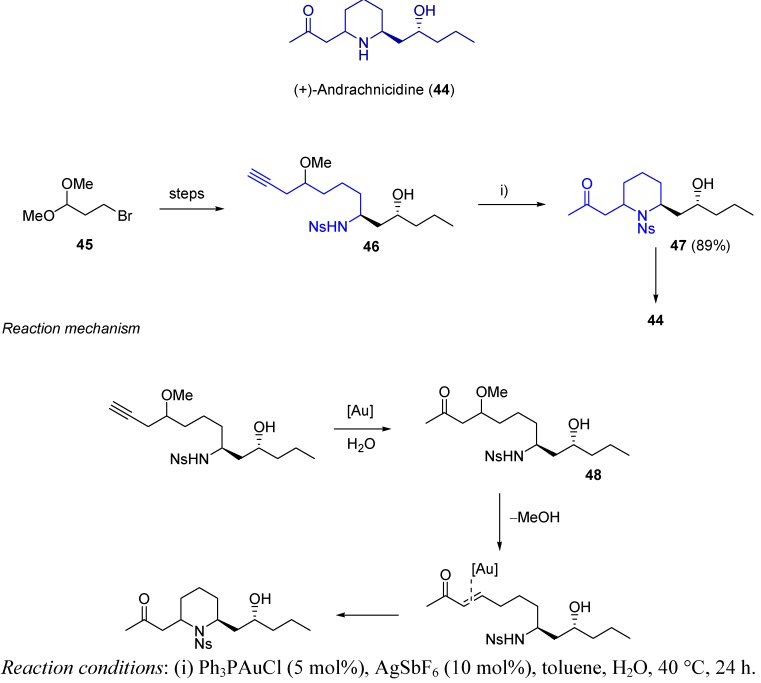
Synthesis of (+)-andrachcinidine.

Another example of gold-based C−N cyclization on alkynol systems for the total synthesis of (+)-andrachcinidine (**44**) has been established [92]. This natural alkaloid receives its name from its natural source, the beetle *Andrachne aspera*, and it has been shown to be an interesting chemical defense agent [93]. The proposed reaction sequence started with commercial ketal **45**, which yielded after six steps the nitrogen-containing alkynol **46**. Gold-catalyzed cyclization of **46** provided the piperidine system **47** as a single diastereomer in 89% isolated yield. The reaction mechanism is proposed to follow a first gold-based alkyne hydration providing ketone **48**. Methoxy group cleavage would then generate the corresponding α,β-unsaturated system, which could undergo nucleophile addition building the expected 6-membered heterocycle (Scheme 15). It is noteworthy that no competition between nitrogen and oxygen attack was found, which would led to the less favoured 8-membered heterocycle.

### 3.2. Cycloisomerization on Alkynediol-Based Systems

Ketals are important key structures, and crucial targets in organic synthesis [94,95,96,97,98]. Fused, bicyclic and spiroketals are recurring motifs in natural compounds, and their preparation is a key step in many total syntheses. In particular, spiroketals represent a structural feature of many biomedically relevant natural and non-natural systems [99,100,101,102]. Several methods have been developed for the synthesis of spiroketals, the most common being perhaps the cyclocondensation of ketone diols [103,104]. Nevertheless, gold catalyzed cycloisomerization on alkynediols has emerged as an efficient strategy to build complex ketal systems in just one step, offering specific advantages. For example, Au-catalyzed cycloisomerization of alkynediols are more exotermic, atom economical, and more compatible than ketones under a number of several reaction conditions. Thus, many groups have recently incorporated the present methodology for the synthesis of several natural compounds and derivatives [105,106,107,108].

**Scheme 16:**
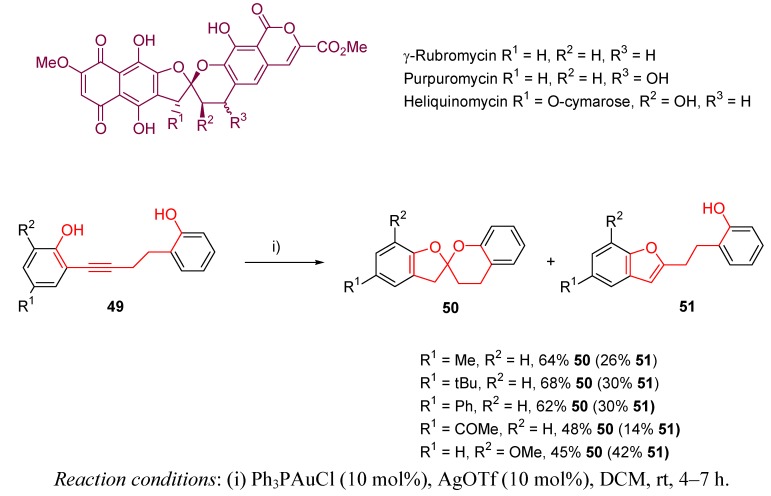
Synthesis of spiroketal motif of rubromycins.

Li *et al.* have described the preparation of the bisbenzannelated spiroketal core of rubromycins [109]. These natural occurring structures exhibit different biological activities, such as inhibition of DNA polymerase, inhibition of the reverse transcriptase of HIV I, or inhibition of DNA helicase [110,111,112,113]. Scheme 16 shows the basic structure motif shared by natural isolated compounds like γ-rubromycin, purpuromycin, or heliquinomycin. According to the described work, easily prepared alkynediols **49** underwent cycloisomerization in the presence of gold catalysis to yield spiroketals **50** with moderate yields, but mainly together with notable amounts of the corresponding benzofuran **51**.

**Scheme 17:**
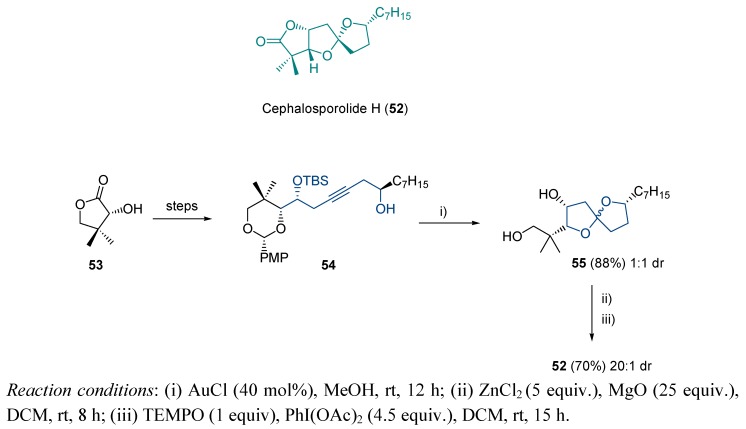
Cephalosporolide H; structure and proposed synthesis.

A more effective spiroketalization process was found for the synthesis of cephalosporolides. Concretely, cephalosporolide H **52** is a natural spiroketal isolated from the culture broth of the marine fungus *Penicillium* sp. This compound presents anti-inflammatory properties by virtue of its inhibitory activity against 3α-hydroxysteroid dehydrogenase [114,115]. Dudley *et al.* developed a method for cephalosporolide total synthesis based on gold-catalyzed spiroketal generation [116,117]. Starting from pantolactone **53**, alkynediol-based system **54** was obtained after a nine step sequence. Gold treatment of **54** yielded the desired structure **55**, with an excellent 88% yield. The main inconvenient of the proposed strategy lied on the obtention of **55** as a 1:1 mixture of spiroketal epimers, although further treatment upon zinc chloride chelation provided the expected isomer in 20:1 dr (Scheme 17).

**Scheme 18:**
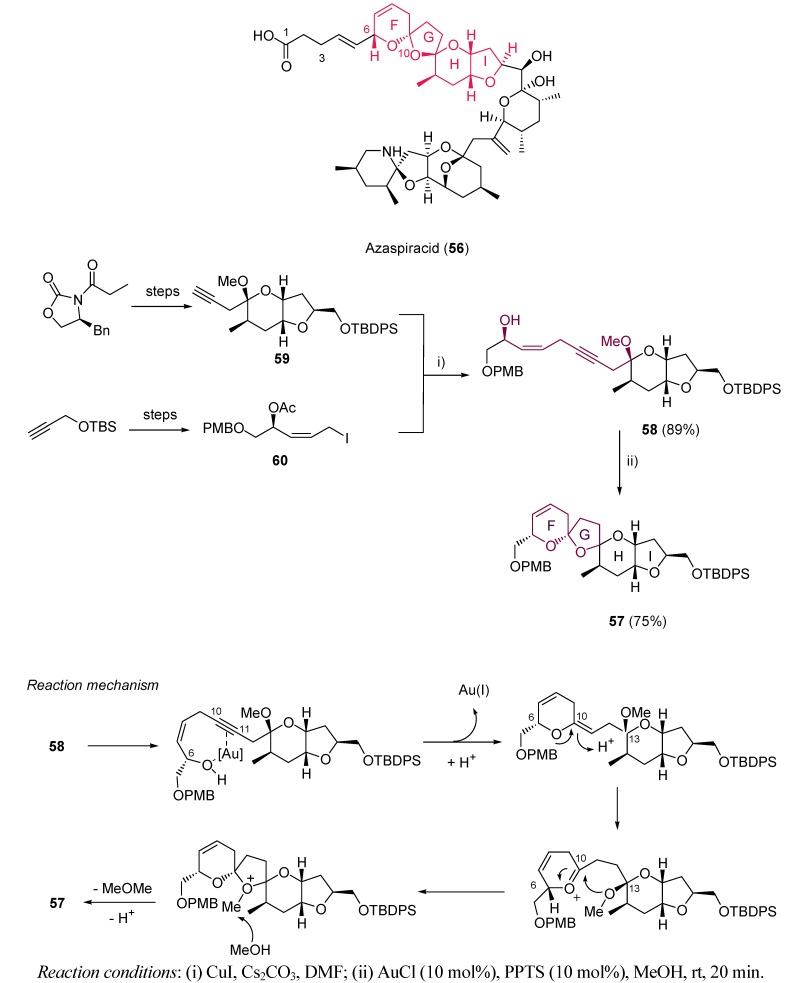
Synthesis of rings **F** and **G** in azaspiracid and reaction mechanism.

Azaspiracid **56** belongs to a family of marine toxins, responsible for human poisoning and diverse chronic effects on liver, pancreas and thymus [118,119]. Its complete structure has been widely studied [120], and several methods for its synthesis have been reported [121,122]. Forsyth *et al*. have reported the synthesis of the **F−I** azaspiracid fragment **57** [123]. In particular, we would like to focus on the construction of **F** and **G** rings by a one step gold-catalyzed spiroketalization. Alkynediol-based system **58** was obtained by coupling of subunits **59** and **60**, prepared from simple precursors. Treatment of **58** with AuCl provided the desired structure **57** with a high 75% yield as a sole isomer. The reaction mechanism is proposed to follow an initial *syn* addition of the C6 hydroxy group and the π-activated gold-alkyne complex to build ring **F**. Protodeauration and protonation of the resultant enol ether at C11 would promote the attack of methoxy oxygen to C10, generating therefore ring **G** (Scheme 18).

**Scheme 19:**
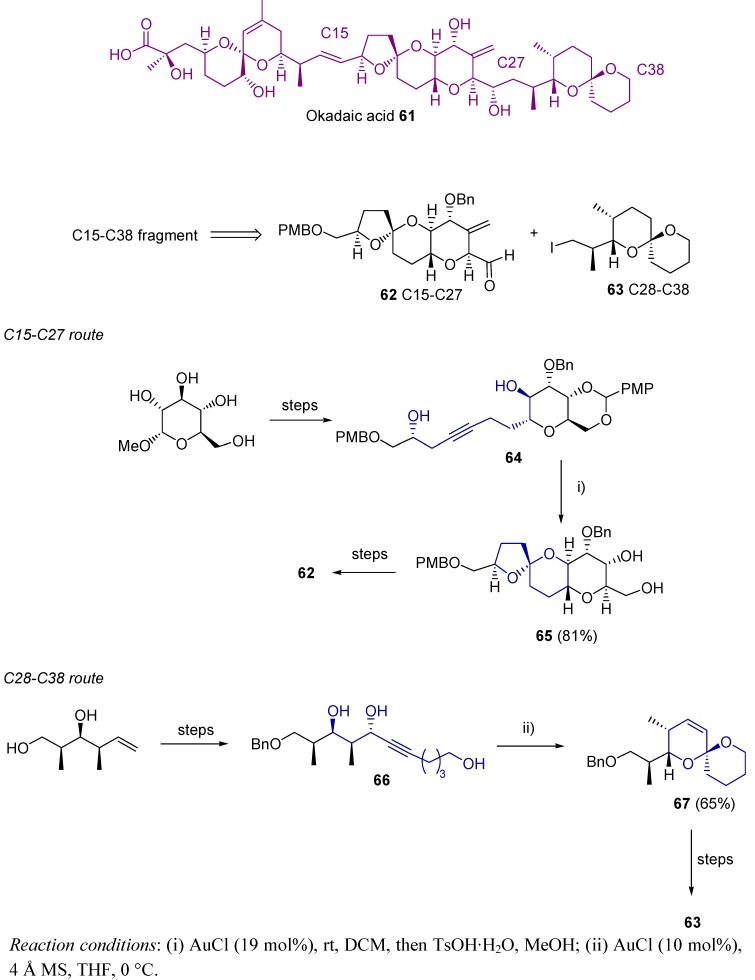
Synthesis of C15-C27 and C28-C38 fragments of okadaic acid.

Okadaic acid **61** is a complex natural structure isolated from marine sponges [124,125]. Its biological activities [126,127,128], together with its attractive chemical structure have attracted much interest among organic chemists. In particular, the presence of several spiroketal motifs in this structure makes it a real challenge from the retrosynthetical point of view. An efficient synthesis of the C15-C38 fragment has been reported, based on the high activity and selectivity of AuCl for the synthesis of spiroketals **62** and **63**, starting from alkynediols **64** and **66** respectively [129] (Scheme 19).

**Scheme 20:**
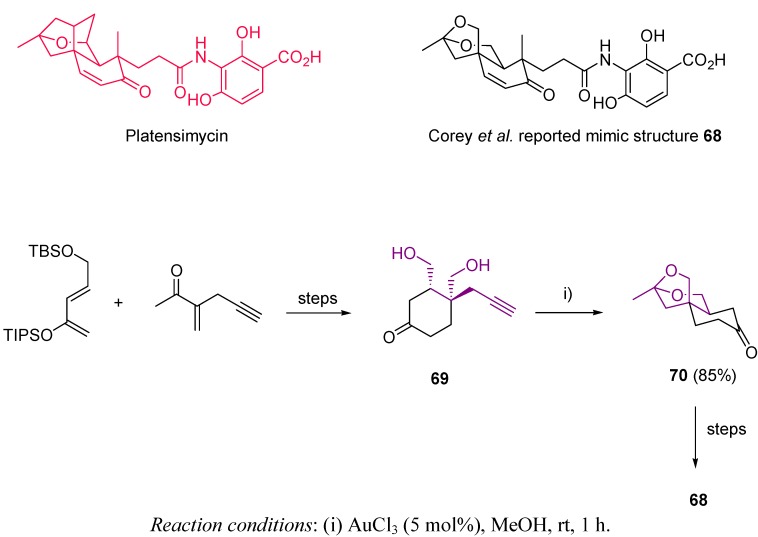
Synthesis of platensimycin-derived structure.

Bridged-bicyclic ketals have been also produced through gold-catalyzed cycloisomerization of alkynediols. Based on platensimycin structure, a natural inhibitor of microbial fatty acid biosynthesis [130,131,132], Corey *et al*. reported the total synthesis of the near-structural mimic **68** [133]. This new structure presents evidence in the literature suggesting excellent antimicrobial properties [134,135]. Thus, easily achieved alkynediol **69** reacted under gold(III) catalysis delivering ketone **70**, which contains the tricyclic core of **68**, with an excellent 85% yield and >98% *ee* (Scheme 20). The route to the desired target is completed in just nine steps, providing a facile and quick methodology to the mentioned bioactive structure.

## 4. Conclusions

In this overview we have collected the most recent advances in gold-catalyzed cycloisomerization of alkynol and alkynediol-based systems for the preparation of natural products and derivatives. This type of process has become an established methodology for accessing a large number of both carbocyclic and heterocyclic structures, containing different sized skeletons. Three to seven-membered carbon rings, such as furan, pyrans, piperidines, and different ketal and spiroketal systems are therefore accessible through this strategy. The reactions discussed herein demonstrate the high synthetic potential of alkynol-based compounds undergoing gold catalyzed cyclization. On the other hand, the efficiency of gold salts and gold complexes have been also documented, allowing mild reaction conditions and great functional group compatibility, specially compared to related thermal or basic rearrangements. In addition, the extremely large number of natural bioactive compounds containing these type of structural motifs, readily available through gold-catalyzed conditions, will certainly provide a renewed and continuous topic of investigation in this field.
